# Objective and Subjective Measures of Midlife Socioeconomic Status and White Matter Hyperintensities

**DOI:** 10.1093/geroni/igaf122.743

**Published:** 2025-12-31

**Authors:** Meredith Phillips, Peter Gianaros, Tae Kim, C Shaaban, Andrea Rosso

**Affiliations:** University of Pittsburgh, Pittsburgh, Pennsylvania, United States; University of Pittsburgh, Pittsburgh, Pennsylvania, United States; University of Pittsburgh, Pittsburgh, Pennsylvania, United States; University of Pittsburgh, Pittsburgh, Pennsylvania, United States; University of Pittsburgh, Pittsburgh, Pennsylvania, United States

## Abstract

The risk of cognitive impairment and dementia is greater among people with lower socioeconomic status (SES). SES is a multifaceted factor encompassing many potential pathways influencing brain structure but is often measured using single variables. Adverse brain features in midlife are associated with faster cognitive aging. We estimated the association between white matter hyperintensity (WMH) volumes and three SES measures: household income, income relative to neighborhood average, and subjective SES in a middle-aged cohort. We fit a series of linear regression models estimating the associations between SES and log-transformed WMHs controlling for age, educational attainment, race/ethnicity. We tested effect measure modification by sex using its interaction with SES measures. Our sample (N = 191) was 40% male, primarily White (84%), and highly educated (88% bachelor’s degree or higher), with a mean age of 42 (SD: 9). We found no associations between total overall WMHs and household income or relative income. Women with higher subjective SES had smaller total overall WMH volume (log-transformed sex*subjective SES β: -0.26, 95% CI: -0.44 – -0.08) but there was no significant association in men. We also found that men with higher subjective SES had larger deep WMH volumes (log-transformed β: 0.55, 95% CI 0.17 – 0.92), while women with higher subjective SES had smaller deep WMH volumes (log-transformed sex* subjective SES β: -0.40, 95% CI: -0.61 – -0.18). Only subjective SES was associated with WMH. We also identified sex differences, as the association between subjective SES and deep WMHs was positive for men but negative for women.

